# Eating Behavior and Women’s Health

**DOI:** 10.3390/nu18010001

**Published:** 2025-12-19

**Authors:** Pasquapina Ciarmela

**Affiliations:** Department of Experimental and Clinical Medicine, Università Politecnica delle Marche, 60126 Ancona, Italy; p.ciarmela@univpm.it; Tel.: +39-0712206270

Nearly 25 centuries ago, Hippocrates proclaimed, “Let food be thy medicine, and medicine be the food”. In the last decade, the importance of diet and the determinants of eating behavior have been re-evaluated.

In this scenario, women’s health can be influenced by complex factors linked to specific conditions, such as adolescence, pregnancy, and menopause. The influence of mealtimes, the quantity of food consumed, food preferences, and food selection can therefore be very complex in women [1,2,3].

This Special Issue of *Nutrients* gathered together ten papers (nine original articles and one review) focused on the most recent investigations into the influence of eating behavior on women’s health, offering valuable insights relevant to gender medicine.

Eating behaviors and food preferences influence food choices, making it difficult to maintain a healthy diet especially in specific life periods such as adolescence and emerging adulthood, or under specific working needs, such as shift work [contributions 1–4].

Globally, binge eating behavior has emerged as a significant public health concern, especially among female adolescents, a population in which concerns around body image can lead to body dissatisfaction and other mental health issues.

A cross-sectional study design was used to evaluate the frequency of binge eating behavior, body shape concerns, and associated factors of 400 female adolescents [contribution 1]. Emerging adulthood (18–30 years) is still a critical period plagued by risks of poor dietary quality and mental health. The study conducted by Al-Bisher and Al-Otaibi revealed a trend of poor adherence to food-based dietary guidelines among Saudi females, with a large proportion also experiencing symptoms of eating disorders [contribution 2]. During adulthood, the shift of schedule may represent a critical impact. Indeed, a study conducted in Wroclaw, Poland, showed that, regardless of their working hours, the diets of midwives are characterized by their low quality [contribution 3]. Similarly, a study conducted in Hong Kong showed that shift work has profound effects on the health and dietary habits of registered nurses. The paper focuses on the need for health care institutions to create and implement nutritional instructions specific to shift workers, helping them to maintain appropriate meal breaks and building a positive work environment in order to enhance eating habits and overall well-being [contribution 4].

General psychological and emotional conditions certainly play an important role in defining eating behavior, so much so that a comorbidity between obesity and depression is often found. A review of the literature endorses the notion that depression and obesity should be treated as one two-dimensional disorder, and that this approach achieves better long-term treatment results. In fact, successful treatment of depression can help in treating obesity, especially in motivating patients to adjust their lifestyle by changing dietary habits and increasing their physical activity, which contributes both changes in body mass index scores and reductions in depressive symptoms. Changes in self-perception, reduced daily stress, and dietary changes, as well as increased physical activity, can contribute both to weight loss and reductions in depressive symptoms [contribution 5].

From the above, it is clear that it is necessary to deploy adequate tools to evaluate dietary decisions. A study conducted among 604 Brazilian adult women supports the use of intuition and deliberation in food decision-making scale as single instrument that is able to measure intuition and deliberation in food decision-making [contribution 6].

Finally, this Special Issue considers specific nutrients that may improve female health generally, or particular conditions such as the premestrual period [contribution 7], the perimenopausal transition [contribution 8], or urinary tract infections [contribution 9].

Specifically, in vitro and in vivo experiments on mice models were employed to examine the use of Lactobacillus helveticus HY7801, which has been tested on premenstrual syndrome symptoms and in relation to sex hormone and inflammatory cytokine overproduction [contribution 7]. The study by Purevdorj and colleagues explored whether porcine placental extract could combat post-menopausal weight gain and lipid imbalances without the side effects of traditional hormone treatments [contribution 8]. Dysbiosis can have an impact on the recurrence of urinary tract infections. The risk factors for recurrent urinary tract infections and lifestyle-induced dysbiosis are potentially controllable, broadening perspectives for new approaches and a changing in the paradigm regarding the treatment of urinary tract infections [contribution 9].

Fish is a food recognized for its beneficial properties, and using fish collagen supplements in daily nutrition may positively influence health and healthy aging. The study by Stelmach-Mardas aimed to assess the serum proteomic changes during fish collagen supplementation in healthy women [contribution 10].

Overall, knowledge on the potential impacts of nutrients and promoting eating behavior on human health and well-being is rapidly improving. This collection includes studies that delve into the benefits of nutrition in terms of prevention approaches and therapies that are specific to the female gender.
